# Supplementary material for: Complexome profiling of the Chlamydomonas psb28 mutant reveals TEF5 as an early PSII assembly factor
Source: Plant Cell. 2025 Mar 17;37(6):koaf055. doi: 10.1093/plcell/koaf055 (PMC12164590; doi:10.1093/plcell/koaf055)
Supplement: koaf055_Supplementary_Data [file koaf055_supplementary_data.zip › psb28_interactive.zip!/plots/intensity/qualityPlots/peptides/_Cre16.g691450.t1.2.html]

### Cre16.g691450.t1.2

Trivial name:   
SeqCoverage [median]: 16.30  
Mass: 72.91 kDa  
  
 

| Sequence | MissedCleavage | Mass [Da] | wtIntensity | muIntensity | Score |
| --- | --- | --- | --- | --- | --- |
| ALGNTDMAR | 0 | 947.45 | 0.00 | 3323.19 | 83.21 |
| FPALVEAK | 0 | 873.50 | 1490.66 | 4571.78 | 91.91 |
| FQTAQSMDKR | 1 | 1210.58 | 1549.52 | 5259.29 | 96.02 |
| GVAPGVALGK | 0 | 867.52 | 1169.30 | 13891.48 | 79.12 |
| IDAIVAR | 0 | 756.45 | 0.00 | 12622.38 | 110.61 |
| IYSLVAGR | 0 | 877.50 | 1966.86 | 11607.11 | 108.24 |
| LAMAYVGEALAYK | 0 | 1398.72 | 1642.29 | 10178.51 | 126.71 |
| MDAAMADNYMAMLSGR | 0 | 1746.72 | 465.01 | 0.00 | 103.88 |
| SGLVSGGLSDGSQRPFK | 1 | 1690.86 | 1183.30 | 6438.50 | 81.66 |
| TGDARFPALVEAK | 1 | 1373.73 | 619.98 | 1791.76 | 56.96 |
| VLVPTTNK | 0 | 870.52 | 0.00 | 3271.76 | 107.43 |
|  |  |  | 10086.9 | 72955.7 |  |
